# Editorial: Resilience Approaches to Promote the Determinants of Health for Indigenous and Other Ethnic Community Youth

**DOI:** 10.3389/fpubh.2020.00338

**Published:** 2020-07-28

**Authors:** Janya McCalman, Robyn Munford, Linda Theron, Jackie Sanders, Roxanne Bainbridge

**Affiliations:** ^1^Centre for Indigenous Health Equity Research, School of Health, Medical and Applied Sciences, Central Queensland University, Rockhampton, QLD, Australia; ^2^School of Social Work, College of Health, Massey University, Auckland, New Zealand; ^3^Department of Educational Psychology, Faculty of Education, University of Pretoria, Pretoria, South Africa

**Keywords:** youth resilience, social determinants of health (MeSH), ethnic communities, indigenous communities, interventions – psychosocial/behavioral

There are currently more than 1.8 billion 10 to 24-year-olds globally; 42% of the world's population is aged under 25 (1). Across different nations, young people have identified consistent priorities: jobs; the chance to start their own businesses; high-quality, relevant education; honest governance; and regional and national security (2). They have also claimed the ideals of “nothing about us without us” and taken central roles in the youth-serving and other sectors to collectively achieve unmet expectations, inclusion, and fair opportunities (3, 4). Supported by youth policies and mainstreaming of youth issues, young people's undeniable strengths and potential as healthy, productive, and engaged citizens is realized (2).

However, 89% of global youth live in less developed countries where barriers to their well-being and the fulfillment of their potential are the highest. Even for those in developed nations, widening ethnicized, and Indigenized inequalities in wealth mean that many young people do not have access to the structural resources that determine health (5). The International Work Group for Indigenous Affairs (6) reported that: "*Indigenous peoples remain on the margins of society: they are poorer, less educated, die at a younger age, are much more likely to commit suicide, and are generally in worse health than the rest of the population*.” Recent decades have seen rising income inequality and inequities, increased effects of armed conflict (7), and accelerating climate change and other environmental shifts, with accompanying risks to human well-being (8, 9). These adversities are associated with worldwide increases in youth mental health disorders, suicide, homicide, obesity, malnutrition, gun violence, road traffic accidents, and precancerous cervical lesions (10). Such issues have impacts through adulthood and intergenerationally since risk exposures that began in adolescence are reflected in 70% of premature adult deaths (11).

For the past 50 years, researchers have noted that young people's experiences of maltreatment, violence, and other traumatic life events produced widely varying outcomes (12). These early findings prompted research about those who, despite risk or adversity, were doing well (13, 14). So arose the concept of resilience as the human capacity to overcome adversity and develop normatively (15). Although early studies foregrounded exploration and definition of the individual assets that make a young person more resilient, current research has expanded that focus to take account of the protective environmental, structural, social, and family factors or “resources” that support coping with adversity (16). Protective factors vary from the proximity of caregivers or other attachment figures during crises (17, 18) to being on-track with education for long-term chronic disadvantages (19). Such protective factors are also known as the social determinants of youth health, defined as the presence (or absence) of income equality and wealth, safe and supportive families, early childhood education and schools, employment with good working conditions, a healthy constructed environment, gender equity, a quality natural environment, and universal healthcare coverage (20). These do not operate independently but mutually influence each other (3, 21).

This Frontiers Research Topic arose from opportunistic discussions between the co-editors at two international resilience research conferences. As delegates from the geographical south, our interests, explored in this Research Topic, lay in understanding the many variations in how young people define and operationalize resilience across cultures and situations. We have also been working to co-design resilience interventions with schools, youth services, and other end-user groups to better support Indigenous and other ethnic community youth, their families, schools and healthcare, and social services to improve youth well-being. In these interventions, young people (as well as school staff, educational support staff, health and youth services and families) participated in decision making to change practices and policies to better meet their needs. We use the ecological systems-focused and culturally sensitive definition of resilience:

“*In the context of exposure to significant adversity, resilience is both the capacity of individuals to* ***navigate*** *their way to the psychological, social, cultural, and physical resources that sustain their well-being, and their capacity individually and collectively to* ***negotiate*** *for these resources to be provided in culturally meaningful ways”* (22).

The four articles in this Research Topic provide evidence of practical ways for strengthening resilience approaches for youth who face adversity. Choudhry et al. explore how young Kalasha people, an Indigenous ethnic and religious minority community in Pakistan, interact with cultural, religious, and other systems. The study provides a first step toward advocating alongside them for a clinical management plan to support resilience and solutions to psychological distress. Jongen, McCalman et al. systematically reviewed the published literature to identify school-based resilience interventions in Canada, Australia, Aotearoa New Zealand, and the United States to improve Indigenous well-being. Indigenous-developed or culturally enhanced interventions ideally incorporated activities to enhance individual assets, community/social resources, and cultural connection and identity. Included were formal programs and the informal championing of resilience by families and communities. Outcomes included improved self-esteem and Indigenous identity, peer support, and social/community connection, community capacity through youth training and leadership opportunities, increased student retention and graduations and reduced school funds spent on external mental health services. Jongen, Langham et al. examined instruments used to measure the resilience of Indigenous adolescents in the same studies. They found only 2/20 administered instruments included items reflecting individual assets, community/social resources, and cultural connection and identity. Finally, Langham et al.'s validation study of an adapted Child and Youth Resilience Measure found results that were inconsistent with its use in other populations. Within a sample of Indigenous Australian boarding school students, rather than a single scale, the instrument measured two separate constructs that captured the sources and expressions of students' resilience.

In conclusion, the papers in this special edition help us to understand better how we can contribute to the call of young native American Sioux and Yacqui man, Victor Lopez-Carmen: “*to deeply and actively consider our actions and their effects on the interests of future generations, taking into account the visions of those before us, and those here now*.” (23). In contributing our legacies, we need to invest in better understanding what resources are needed to support the resilience of young people across cultures and contexts and advocate to improve their equitable access to these resources, thereby enhancing capabilities and well-being toward a positive inter-generational here and after.

## Author Contributions

JM, RB, and LT conceived of the Research Topic concept. JM developed the editorial draft. All authors discussed and contributed to the final manuscript.

## Conflict of Interest

The authors declare that the research was conducted in the absence of any commercial or financial relationships that could be construed as a potential conflict of interest.
